# Extracellular Vesicles in Acute Kidney Injury and Clinical Applications

**DOI:** 10.3390/ijms22168913

**Published:** 2021-08-18

**Authors:** Sekyung Oh, Sang-Ho Kwon

**Affiliations:** 1 Department of Medical Science, College of Medicine, Catholic Kwandong University, Incheon 22711, Korea; ohskjhmi@cku.ac.kr; 2 Department of Cellular Biology and Anatomy, Medical College of Georgia, Augusta University, Augusta, GA 30912, USA

**Keywords:** acute kidney injury, extracellular vesicles, exosomes, microvesicles, apoptotic bodies, biomarkers, liquid biopsy, mesenchymal stem cells, injury repair

## Abstract

Acute kidney injury (AKI)––the sudden loss of kidney function due to tissue damage and subsequent progression to chronic kidney disease––has high morbidity and mortality rates and is a serious worldwide clinical problem. Current AKI diagnosis, which relies on measuring serum creatinine levels and urine output, cannot sensitively and promptly report on the state of damage. To address the shortcomings of these traditional diagnosis tools, several molecular biomarkers have been developed to facilitate the identification and ensuing monitoring of AKI. Nanosized membrane-bound extracellular vesicles (EVs) in body fluids have emerged as excellent sources for discovering such biomarkers. Besides this diagnostic purpose, EVs are also being extensively exploited to deliver therapeutic macromolecules to damaged kidney cells to ameliorate AKI. Consequently, many successful AKI biomarker findings and therapeutic applications based on EVs have been made. Here, we review our understanding of how EVs can help with the early identification and accurate monitoring of AKI and be used therapeutically. We will further discuss where current EV-based AKI diagnosis and therapeutic applications fall short and where future innovations could lead us.

## 1. Introduction

Unceasingly ridding the blood of metabolic waste and reabsorbing essential molecules into our body, the kidney controls body fluid volume, osmolarity, acid–base balance, electrolyte concentration, and toxic substance removal. In playing such vital roles, however, the kidney is often exposed to various endogenous or exogenous insults that can disturb this exquisitely maintained body-fluid homeostasis [1,2]. One such disordered clinical condition, acute kidney injury (AKI) is characterized by a sudden decline in normal function, resulting from injury to cells in the nephron [1,3]. Prevalent in hospitalized patients, AKI poses a serious clinical problem worldwide with high mortality and morbidity [1,3]. Developing a reliable AKI diagnosis and prognosis, therefore, must be actively pursued [4,5].

Fluids such as blood and urine hold numerous membrane-bound, nanosized vesicles that are secreted by many cell types [6,7,8]. Such extracellular vesicles (EVs) contain a set of macromolecules that reflect the properties of the cells from which they are generated [6,7,8]. Shielded by the vesicle membrane from the hydrolyzing activity abundant in biological fluids, EV cargo macromolecules can also elicit biologically relevant responses when transported to proximate or remote target cells for uptake [6]. These characteristics of EVs have thus been exploited not only to examine the physiological or pathological states of EV-producing cells in varied biological contexts but also to deliver therapeutically potent, bioactive molecules, enclosed within EVs, to desired target cells [9]. Accordingly, recent advances on AKI research have also benefited from developments in biomarker discovery and engineering such as using EVs [10].

In that regard, we summarize our current understanding of how AKI is variously diagnosed; how EVs help us come up with better diagnoses and prognoses, apply EV knowledge to ameliorate AKI, and prepare EVs for translational and therapeutic purposes. We further discuss challenges associated with isolating and analyzing EVs.

## 2. A Growing Demand for a Better Diagnosis of Acute Kidney Injury (AKI)

A considerably large share of mortality and morbidity around the world is attributed to impaired kidney functions, and AKI is a dire form of such renal failure manifested as an abrupt reduction of the normal kidney function, resulting from an injury to cells in the nephron. A wide variety of intrinsic and extrinsic factors such as oxidative stress and drug abuse are known to cause AKI to develop within days [11]. It poses a serious clinical problem worldwide as it afflicts 10–15% of patients admitted to a hospital and more than 50% of patients in an intense care unit [12,13,14].

Even though the kidney is thought to have some ability to regenerate itself after injury [2], most AKI patients do completely recover but proceed to develop tubulointerstitial fibrosis and chronic renal inflammation, ultimately leading to chronic kidney disease (CKD) [11,15,16,17]. Moreover, AKI often complicates the clinical courses of the patients by requiring dialysis and prolonged hospitalization leading to high morbidity and mortality [3]. Therefore, early detection of AKI with accurate staging is crucial for selecting the proper treatment, which is currently limited to supportive care to prevent AKI from becoming aggravated.

AKI is typically diagnosed and staged by measuring changes in serum creatinine and urine [18,19]. Serum creatinine levels can be found elevated in AKI due to a diminished glomerular filtration rate (GFR), but they cannot sensitively report on damage to the kidney. In fact, since AKI predominantly affects the tubules in the nephron, such glomerular measurements as GFRs and their proxy, serum creatinine levels, would only indirectly indicate an injury in the tubule [5]. Thus, only after either GFR declines substantially or a tubular injury progresses considerably, will serum creatine levels noticeably rise. Importantly, alterations in serum creatine levels, per se, cannot account for how GFR declines or how AKI occurs [20]. Likewise, reduced urine output (oliguria), which is frequently found in AKI patients, can neither notify us of injury incidence promptly nor inform us of the underlying process.

Using such indirect AKI indicators as serum creatinine levels and urine output could further convolute AKI diagnosis and prognosis, for these measurements could easily be influenced by a patient’s age, gender, diet, muscularity, hydration, and medication profile [21,22]. These current diagnostic limitations have thus prompted studies to discover and evaluate more sophisticated molecular biomarkers that would enable a timely diagnosis accurate, precise staging, and a better prognosis [21,22].

## 3. Uncovering AKI Biomarkers

Advances in state-of-the-art mass spectrometry analysis and other related molecular methodologies have led to uncovering several AKI molecular biomarkers. All these efforts aimed at discovering such candidate macromolecules would enable sensitive, early detection; help predict clinical courses of treatment; and facilitate therapeutic decisions [23]. Consequently, while not yet globally commonplace, select biomarkers have entered the clinical use stage in some countries. For example, l-FABP (liver-type fatty acid-binding protein) and NGAL (neutrophil gelatinase-associated lipocalin) are approved for use in Japan and Europe, respectively. The United States Food and Drug Administration (FDA) has authorized the use of a combination of TIMP-2 (tissue inhibitor of metalloproteinase-2) and IGFBP-7 (insulin growth factor binding protein-7) [24]. The FDA has also permitted a panel of such urinary biomarkers as KIM-1 (kidney injury molecule-1), Cystatin C, Clusterin, and β-2-microglobulin for preclinical use to detect nephrotoxicity [25]. Notably, many of these molecular AKI biomarkers can be acquired and analyzed in a non-invasive manner, for these are released into urine in response to injury [5].

Alongside such a procedural advantage, these AKI biomarkers can also inform us of the site of injury in the nephron, albeit roughly. For example, KIM-1, l-FABP, IGFBP-7, and TIMP-2 are known to come from the proximal tubule; UMOD (Uromodulin, also known as Tamm Horsfall Protein) from the loop of Henle; and NGAL from the distal tubule and the collecting duct [5]. As diverse tissue responses likely accompany AKI in the kidney, studies have also discovered biomarkers that report inflammation (e.g., MCP-1, OPN, and IL-18), fibrosis (e.g., TGF-β, and PIINP), and repair (e.g., EGF, YKL-40, and OPN) [5] in addition to those that indicate injury. Although these biomarkers can account for the presence or progression of injury in a manner rather non-specific to AKI, the information gained from them can complement what is attainable from using injury-reporting urinary AKI biomarkers.

The discoveries of the biomarkers described above have already shed light on improving the diagnosis and prognosis of AKI. The appropriate practice of these biomarkers can help predict the progression of AKI to CKD, discern kidney dysfunction and injury, and manage the clinical courses of AKI patients. It can also guide the selection of homogeneous patient groups to plan better clinical trials aimed at AKI. Nonetheless, one should still be cautious when interpreting the performance of these biomarkers, for their temporal profiles can fluctuate with such factors as time after injury, baseline renal injury, and subclinical kidney disease [20,26]. Hence, while improving the implementation of the known biomarkers, recent investigations have moved on to an alternative biomarker discovery basis, namely, extracellular vesicles in body fluids, for these likely hold and concentrate potentially informative macromolecules [27,28].

## 4. Extracellular Vesicles as a Basis for Biomarker Discovery

The extracellular milieu consists of a myriad of macromolecule species that are produced and released by many cell types. While some macromolecules disperse only locally from the source cell after release, others can travel to distant locations, complicating tracing of the producing cell. Furthermore, certain macromolecule species, such as albumin and immunoglobulin in the blood plasma, are known to dominate the molecular profile of the body fluid [29], making the identification of other significant macromolecules at low levels challenging. Moreover, the hydrolyzing activity that degrades macromolecules with varying susceptibility abounds in the extracellular milieu [29]. Hence, complexity in the molecular composition of the extracellular space and the instability of extracellular macromolecules have impeded efforts to discover biomarkers in body fluids [29]. In this regard, the nanosized, membrane-bound extracellular vesicles (EVs) have intrigued researchers in various fields [30,31,32], for these may not only concentrate a subset of extracellular macromolecules to reduce the molecular population complexity but also shield them from hydrolyzing activity to permit consistent recovery.

Generated and released by most cell types in our body, EVs can be recovered from a variety of body fluids, including blood, urine, saliva, and cerebrospinal fluid [30,31,32]. Earlier studies revealed that some cell types such as erythrocytes can remove obsolete membrane proteins by excreting them on EVs, thereby remodeling intracellular membranes [33,34]. Adding to such a somewhat simple cell pruning role, recent studies have also ascribed numerous intercellular communication roles to EVs. Thus, they are now thought to mediate biological processes between cells, ranging from body patterning and immune response to tissue repair and tumor development [35,36], by transporting a set of proteins and nucleic acids to target cells.

**Figure 1:**
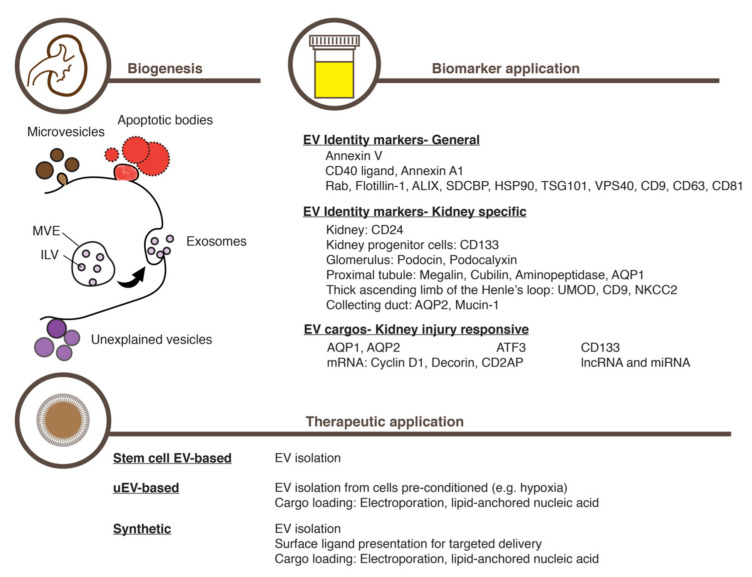
Generated by multiple mechanisms, extracellular vesicles (EVs) carry a set of macromolecules that reflect the normal and the pathological (e.g., acute kidney injury) states of the producing cells. EVs can also be engineered to deliver therapeutically potent, bioactive macromolecules to desired target cells. MVE, multivesicular endosome. ILV, intralumenal vesicle, uEV, urinary EV.

Cell biological studies on EV biogenesis mechanisms have broadly categorized EVs into three major classes (Figure 1). The smallest exosomes (30–100 nm in diameter), are known to emerge through intricate intracellular membrane trafficking. The formation of the multivesicular endosomes (MVE, also known as the multivesicular bodies, MVB) is the key step in exosome-generating membrane trafficking (Figure 1) [37,38]; that is, the membrane of the late endosomes invaginates and pinches off, giving rise to numerous intraluminal vesicles (ILVs) inside the resulting outer surrounding vesicle, MVE. A specific set of macromolecules are thought to board ILVs during this process. Charged with these constituents, ILVs are eventually secreted as exosomes when the outer MVE membrane fuses to the plasma membrane [37,38]. Numerous factors are known to regulate exosome generation and release, including membrane trafficking proteins [39,40,41], the Rab family of small GTPases [42,43], the ESCRT-III (the endosomal sorting complex required for transport-III) machinery [44], the Sirtuin family of deacetylases [45,46], the tetraspanin family of membrane proteins [47], and certain lipids such as ceramide [48].

In contrast to this complexity of exosome biogenesis, the second EV class, microvesicles, is known to develop through a simpler process: the outward budding of the plasma membrane (Figure 1) [31,49]. While larger in size (100–1000 nm in diameter) than exosomes, microvesicles also pack and release a specific set of macromolecules as cargo. The third EV class, apoptotic bodies, are known to form when the membrane of an apoptotic bleb wraps many parts of the cell in relatively large vesicles of up to 4000 nm in diameter (Figure 1) [50]. To make matters more complex, recent studies are still uncovering novel EV classes as well as subclasses of a known EV type [51,52,53,54].

Existing in a continuum in size, density, and content [51], discerning these vesicles in the extracellular space seems difficult, however. The term EV is instead suggested to refer to all these vesicle types in the aggregate [30,31,32]. Nevertheless, one can still distinguish individual EV classes by using characteristic molecular markers (Figure 1). For example, Annexin V appears on the membrane of apoptotic bodies [52,55]; and CD40 ligand [56,57] and Annexin A1 [52] on that of medium-to-large EVs. Small EVs such as exosomes, on the other hand, are known to contain Rab proteins: Flotillin-1, the ESCRT-III machinery, ALIX, TSG101, and VPS4. Also, tetraspanins such as CD9, CD63, and CD81 are known to curve biological membranes, thereby favoring their existence on small EVs [31]. Furthermore, lipid composition also varies among EV classes, with sphingolipids such as sphingomyelin and ceramide existing mostly on small EVs [58].

EVs can deliver donor cell information to either proximate or remote cells. Not only individual macromolecules but also a subcellular organelle in its entirety such as a mitochondrium can be transferred via an EV to a target cell [59,60]. Several mechanisms, such as endocytosis and membrane fusion are known to unload EV contents in the target cell interior. The delivered macromolecules can, in turn, induce phenotypic alterations of the target cell. For example, certain transported messenger RNA (mRNA) molecules are translated into proteins, inducing de novo expression of the proteins in the target cell. On the other hand, microRNAs (miRNA) are also delivered via EVs to recipient cells to reduce the level of the target mRNAs, thereby decreasing their expression [61,62,63,64]. Phenotypic alterations can also be induced by unpacking proteinaceous EV cargos in the target cell interior. For example, proteins transported via EVs can confer the ability to either promote or suppress immune responses upon the receiving cell [65].

## 5. Urinary Extracellular Vesicles Containing Information on the Kidney

Urine fascinates EV researchers, for urinary EVs can be isolated and analyzed economically and conveniently in a non-invasive manner outside our body [66]. Moreover, mass spectrometry studies have found that urinary EVs can serve as an excellent source for biomarkers [67]. A majority of urinary EVs are released apically from the epithelial cells facing the tubular lumen. Urinary EVs can also come from other parts of the kidney that drain urine such as the collecting duct cells, podocytes and other glomerular, progenitor, and infiltrated inflammatory cells [68,69,70,71,72].

Studies have revealed that urinary EVs carry typical exosome markers, including tetraspanins (CD9, CD63, and CD81), Flotillin-1, HSP70, and ALIX (Apoptosis-linked gene-2-interacting protein X), among others (Figure 1) [73]. Moreover, urinary EVs also express markers that denote the kidney origin such as CD24 (Figure 1) [74]. Other macromolecules in urinary EVs can further inform us of where in the kidney individual EVs originated, for example: Podocin and Podocalyxin come from podocytes in the glomerulus [75]; Megalin, Cubilin, Aminopeptidase [76], and Aquaporin-1 (AQP-1) come from the proximal tubule [77]; UMOD, CD9, and NKCC2 (Type 2 Na–K–2Cl co-transporter) are from the thick ascending limb of the Henle’s loop; and AQP-2 (Aquaporin-2) and Mucin-1 are from the collecting duct (Figure 1) [71,78]. Moreover, the urinary EVs coming from renal progenitor cells can also be identified by detecting CD133 expression (Figure 1) [79,80]. In addition to these proteinaceous markers, urinary EVs harbor RNA molecules. While the majority of these RNAs are ribosomal and non-encoding [81], miRNAs and protein-coding mRNAs in urinary EVs are also implicated in regulating kidney functions (Figure 1) [82].

In addition to simply reflecting the status of the producing cells, urinary EVs may also mediate information exchange between cells in the nephron [83]. Since urine flows unidirectionally, EVs produced by proximal tubule cells may travel down the road along the tubule to induce phenotypic alterations in cells in the distal tubule and the collecting duct [84]. For example, EVs released from the proximal tubule cells treated with a dopamine receptor agonist can reduce radical production in the recipient distal tubule cells, thereby spreading an anti-inflammatory response across the nephron [84].

## 6. Urinary Extracellular Vesicles in Acute Kidney Injury and Recovery

Given the convenience and feasibility in uncovering biomarkers, urinary EVs are revealing several potentially useful macromolecules that report on AKI. Unlike serum creatinine levels and urine output, these macromolecules carried by urinary EVs appear to mirror promptly the injured state of the kidney, enabling more accurate disease staging, diagnosis, and prognosis (Figure 1). This advantage is exemplified by the ATF3 (Activating transcription factor 3) protein in urinary EVs, the level of which substantially increases in response to an acute injury induced by either cisplatin or ischemia-reperfusion (Figure 1) [69]. Interestingly, the ATF3 protein level increases in response to AKI specifically in urinary EVs but not in the urine per se [69], demonstrating the utility of EVs in concentrating and stabilizing extracellular macromolecules. Moreover, the rise of the ATF3 level in urinary EVs precedes that of serum creatinine, with the elevated level staying for 24–48 h [69], which suggests that timely registration of the damage can be achieved by measuring ATF3 protein levels in urinary EVs. The level of mRNAs encoding ATF3 was also elevated in urinary EVs after AKI induction [85]. Other potentially useful proteins in urinary EVs, the levels of which increase in response AKI prior to the rise in serum creatinine, include Fetuin-A [86] and NGAL [87]. On the other hand, the level of AQP-1 in urinary EVs, a marker of the proximal tubule, declines in response to ischemia–reperfusion-mediated acute kidney injury in rats as well as kidney transplantation in human patients [77].

Importantly, the content of urinary EVs changes while the damaged kidney is recovering from AKI as shown by phenotypic alterations in the nephron (Figure 1) [82]. For example, in rat kidney such miRNA cargo as miR-16, miR-24, and miR200c are released right after an acute ischemia-reperfusion injury [82]. In the following early recovery stage, a different set of miRNAs such as miR-9a, miR-141, miR-200a, miR-200c, and miR-429, which commonly target Zeb1/2 mRNA, are released, thereby engaging TGF-β-mediated fibrosis in the nephron during recovery from the injury [82]. The induced TGF-β signaling, in turn, causes another set of miRNAs, miR-125a and miR-351, to be released [82].

The CD133-expressing progenitor cells in the kidney epithelium play key roles not only in continuous renewal of the kidney epithelium but also in recovery from AKI through regeneration of the damaged tissue (Figure 1) [88,89,90,91]. The number of CD133-expressing cells increases in response to an acute damage of the kidney [92,93]. Thus, the increase in cell number likely accounts for the increase in the number of CD133-containing EVs in urine [93]. These CD133-containing urinary EVs are thought to engage a certain gene expression program to promote cell cycle progression and survival, possibly transporting such cargo as Cyclin D1 and Decorin mRNAs (Figure 1) [94].

## 7. Extracellular Vesicles from Mesenchymal Stem Cells for Treating AKI

The utility of EVs goes beyond just cataloguing the contents and identifying informative molecules that report on disease features and progression. To keep abreast of such basic biological endeavors, various translational research fields also use EVs as a vehicle to deliver therapeutic molecules to specific, desired target cells. Accordingly, a great deal of research has explored the possibility of ameliorating renal diseases such as AKI by making use of EVs released from a homogeneous cell source: principally, mesenchymal stem cells (also known as mesenchymal stromal cells, MSCs), that can supply EVs quite consistently.

Derived from a variety of tissues such as bone marrow, adipose tissue, and the umbilical cord, MSCs commonly express such surface markers as CD44, CD73, CD90, CD105, and CD146 [95] and have been used in the treatment of various diseases, mainly through helping tissue regeneration [96]. However, attempts to implant MSCs stably in a targeted tissue have proven difficult to achieve [97,98,99,100,101,102]. Instead, it has become evident that a conditioned culture medium of MSCs can have therapeutic effects comparable to those of the transplanted cells, themselves [103,104,105]. Among the conditioned culture media of MSCs, EVs were proven to have therapeutic potential for a variety of diseases, including AKI (Figure 1) [106,107,108,109]. A recent meta-analysis of 31 independent preclinical rodent AKI models confirmed the therapeutic potential of MSC-derived EVs in treating AKI [110].

MSC-derived EVs appear to exert therapeutic effects through conferring upon the kidney cells the ability to resist the apoptosis of healthy epithelial cells [111,112], stimulate the recovery process such as cell-cycle reentry [111,112], and suppress inflammatory responses [107]. These effects are presumably based on transferring certain critical cargo molecules in the MSC-derived EVs to the injured tissue. In fact, delivering such factors as the IGF-1 (Insulin-like growth factor 1) protein and mRNA via MSC-derived EVs are well documented for their ability to regenerate a damaged kidney [113]. In addition, miRNAs transported by MSC-derived EVs may also play a crucial role in eliciting therapeutic effects for AKI. In MSCs, the knockdown of Drosha, an essential component in primary miRNA processing [114], can prevent therapeutic effects [115].

While EVs from a variety of MSC sources can assist recovery from AKI [116,117,118,119,120,121,122], this complexity of cell origin and the varied culture conditions may confound the efforts to purify or enrich EVs from the conditioned culture medium. Co-isolating substances present in the conditioned culture medium may lead to a wrong conclusion about the therapeutic potential expected from the EVs. Moreover, the adequate effective amount, route of delivery, and biodistribution of MSC-derived EVs need to be determined more definitively [123,124]. Even so, EVs derived from MSCs provide us with several advantages such as low immunogenicity [125,126,127], high biological tolerance [128], and rapid internalization into target cells [129].

## 8. Preparing Extracellular Vesicles for Treating AKI

When using EVs to treat diseases such as AKI, one should make careful decisions about where to obtain EVs, how to load EV cargos, and how to enrich EVs from heterogenous mixtures with consistent quality. While several MSC types may produce EVs of comparable effects, those that have similar characteristics to the renal system will likely be better at treating AKI. In the same vein, EVs derived from either renal progenitor cells or their differentiation progenies may excel those from other cell sources (Figure 1). The recent development of the protocol to differentiate human pluripotent stem cells into the kidney organoid could be utilized to generate a large quantity of homogenous EVs (Figure 1) [130]. Since pluripotent stem cells can be induced from somatic cells obtainable from the patient who would later want to receive an EV therapy, the EVs prepared using this approach could avoid many obstacles such as unwanted immune responses and ethical issues. Moreover, CRISPR/Cas9-mediated genome editing has shown its utility by precisely altering the genome of the pluripotent stem cells and, subsequently, that of their differentiated progenies in diverse research fields [131]. Employing this approach in AKI studies, therefore, would not only reveal definitively how individual genetic factors can modulate EVs and contribute to amelioration of AKI but also enable production of EVs tailored to the genetic make-ups of individual patients.

On the other hand, a great deal of engineering effort is being made to improve EV cargo loading and EV delivery to target cells (Figure 1). For example, the MSCs engineered to express the miRNA Let7c were found to localize preferentially to the injured kidney, resulting in an efficient transferring of Let7c in EVs to the damaged tissue to repress expression of the genes involved in TGF-β-mediated fibrosis [132]. EV engineering studies targeting other organs illuminates how to address AKI with engineered EVs. These include a fusion of the exosome membrane protein LAMP2B to the neuron-specific RVG peptide 3 to improve neuronal delivery [133], an enhanced stable retention of exosomes in circulation by expressing CD47 in MSCs [134], and the development of a novel molecular platform called ARMS (Arrestin domain containing protein 1-mediated microvesicles) to improve cargo packaging and delivery [135]. In this vein, a recent development of the specific delivery of erythrocyte-derived EVs charged with therapeutic small interfering RNAs (siRNAs) to the damaged kidney with AKI is remarkable [136]. The key to this feat was the development of synthetic peptides that bound to the AKI biomarker KIM-1 [136]. Expressing the peptides on the EVs derived from erythrocytes delivered the EVs charged with therapeutic siRNAs specifically to the AKI kidney, ameliorating tubulointerstitial inflammation and fibrosis [136].

In addition to utilizing intrinsic cell mechanisms of EV cargo loading, macromolecules of potential therapeutic values can also be introduced by incubating with isolated EVs in a test tube condition (Figure 1). Multiple methods have been explored to achieve this, including simple incubation, electroporation, and saponin-assisted loading [137]. Among these, simple incubation appears to work well for loading hydrophobic compounds [138] and proved its utility in delivering the organic compound curcumin [139]. On the other hand, electroporation was shown to be useful for loading exogenously prepared miRNAs [140] and long non-encoding RNA [141]. Moreover, the saponin-assisted encapsulation showed an enhanced delivering efficiency of the CRIPR/Cas components to the target cells, thereby facilitating precise genome editing [142].

## 9. Challenges in Isolating and Analyzing Extracellular Vesicles

Both basic biological and translational EV studies critically rely on methods to isolate EVs with high purity and consistency. While a variety has been developed, each has relative strengths and weaknesses. One should, therefore, understand what each method can offer and how to use them appropriately.

The most widely accepted method for EV isolation is multiple rounds of differential centrifugation and pelleting exosomes at the last ultracentrifugation step [143,144]. While this approach promises to isolate EVs at the purest level among the current methods, the whole process is laborious, time consuming, and limited in scalability. Also, ultrahigh gravitational forces can also cause EVs to aggregate, thereby potentially impairing their integrity [145]. Notably, ultracentrifugation of the conditioned culture medium may also pellet a substantial number of particles unrelated to EVs. In preparing EVs from a molecularly complex fluid, therefore, one might also consider further fractionating the EV fraction of ultracentrifugation by using another method such as density gradient centrifugation [146].

Separating particles by size is another approach for isolating EVs at a relatively pure level with varying scalability. This includes methodologies such as relying on either ultrafiltration concentration [147] or tangential flow filtration [148], often followed by size exclusion chromatography to purify EVs. In addition, various affinity capturing methods are widely used to isolate EVs, such as immunoaffinity capturing with anti-EpCAM and anti-CD63 antibodies [149]. However, immunoaffinity methods can only capture a subset of EVs, which express the antigen on the EV surface [150,151]. A similar affinity capturing method uses the interaction between phosphatidylserine on the surface of some EVs and Tim4 (T cell immunoglobulin and mucin domain protein 4) on capturing magnetic beads [151]. A recently developed affinity-based capturing method using lipid nanoprobes holds great promise in that it can isolate highly pure EVs with a much shorter processing time relative to differential centrifugations and with scales conducive for high-throughput screening and analysis [152]. On the other hand, polymer-mediated precipitation also appears useful for isolating EVs, particularly to confirm EV biomarker performance [153]. The high yield of EV isolation by this method, however, comes at a cost in that it can also precipitate a large amount of particles unrelated to EVs [154].

To be used in clinics, EVs must lack contaminating xenogeneic substances, which can be avoided through culturing the EV-producing cells in a defined culture medium [9]. EVs prepared for therapeutic purposes should also pass a high purity threshold to preserve the physical and functional integrity [155]. Moreover, for consistent production, EV isolation scales should be adjustable without compromising quality [155]. To this end, EV manufacturing processes are being devised by combining the advantages that different EV isolation methodologies can provide. For example, an EV isolation procedure with tangential flow filtration and size exclusion chromatography in succession might promise high purity, scalability, and reproducibility.

## 10. Conclusions

Although AKI remains as a serious clinical problem that threatens the lives of countless patients worldwide, the recent development in biomarker discovery and regenerative therapy based on EVs is rapidly advancing our understanding and treatment of this dreadful disease. However, we still need to upgrade our EV biomarker inventory by further discovering and detailing where the disease-reporting macromolecules are generated in the nephron, when and how each of these is released via EVs, and how the levels of these biomarkers change over time. This new information should not only enable a more precise diagnosis and prognosis of AKI patients, but in turn help us design better therapeutic applications of EVs. The continuous methodological innovations in cell culturing, vesicle isolation, cargo loading, specific delivery to the damaged kidney will gradually solve many obstacles remaining in utilizing EVs with adequate quantity and quality for AKI therapy. Rapid developments in precise genome editing with the CRISPR/Cas system and in patient-derived kidney organoids from the induced pluripotent stem cells will further accelerate our endeavors to treat this hard-to-cure disease.

## Data Availability

Not applicable.
